# Supplementary material for: Mechanisms governing the pioneering and redistribution capabilities of the non-classical pioneer PU.1
Source: Nat Commun. 2020 Jan 21;11:402. doi: 10.1038/s41467-019-13960-2 (PMC6972792; doi:10.1038/s41467-019-13960-2)
Supplement: Supplementary file 7 — Source data [file 41467_2019_13960_MOESM7_ESM.zip › Source_Data/Figure6/Figure6B_MotifScanOutput/FLI1_PU.1/homerResults/motif34.similar.html]

motif34

## Information for motif34

T
C
A
G
T
G
A
C
T
C
A
G
A
G
T
C
G
T
C
A
A
G
C
T
A
C
T
G
A
G
T
C
A
C
T
G
G
A
T
C
C
G
T
A
  
Reverse Opposite:  

G
C
A
T
C
T
A
G
T
G
A
C
T
C
A
G
A
G
T
C
T
C
G
A
C
A
G
T
A
C
T
G
A
G
T
C
A
C
T
G
A
G
T
C
  

|  |  |
| --- | --- |
| p-value: | 1e-29 |
| log p-value: | -6.768e+01 |
| Information Content per bp: | 1.736 |
| Number of Target Sequences with motif | 275.0 |
| Percentage of Target Sequences with motif | 2.76% |
| Number of Background Sequences with motif | 401.1 |
| Percentage of Background Sequences with motif | 1.10% |
| Average Position of motif in Targets | 99.1 +/- 56.4bp |
| Average Position of motif in Background | 92.2 +/- 65.6bp |
| Strand Bias (log2 ratio + to - strand density) | 0.1 |
| Multiplicity (# of sites on avg that occur together) | 1.12 |
| Motif File: | file (matrix) reverse opposite |

### Similar de novo motifs found

|  |  |  |  |  |  |  |  |
| --- | --- | --- | --- | --- | --- | --- | --- |
| Rank | Match Score | Redundant Motif | P-value | log P-value | % of Targets | % of Background | Motif file |
| 1 | 0.987 | T C A G T G A C T C A G A G T C G T C A A C G T A C T G A G T C A C T G G A T C T G C A T A C G | 1e-27 | -63.042159 | 2.74% | 1.13% | motif file (matrix) |
| 2 | 0.987 | A T G C A G C T C T A G T G A C C T A G A G T C T G C A C A G T A C T G A G T C A C T G G A T C T C G A | 1e-26 | -60.707094 | 2.34% | 0.90% | motif file (matrix) |
| 3 | 0.942 | T A G C C A T G T G A C G C T A C G A T A C T G T A G C A C T G A G T C T C G A | 1e-23 | -53.345388 | 2.22% | 0.90% | motif file (matrix) |
| 4 | 0.900 | C T A G G A T C T C A G A G T C G C T A G C A T T A C G G A T C A C T G | 1e-22 | -50.968671 | 3.14% | 1.54% | motif file (matrix) |
